# Maternal Mortality: Causes, trends and delays in care at Tertiary care hospital, Pakistan

**DOI:** 10.12669/pjms.41.2.9974

**Published:** 2025-02

**Authors:** Tayyiba Wasim, Saira Yunus, Gul-e-Raana, Anaab Wasim

**Affiliations:** 1 Tayyiba Wasim, FCPS. Department of Obstetrics and Gynaecology, Services Hospital, Lahore, Pakistan; 2 Saira Yunus, FCPS. Department of Obstetrics and Gynaecology, Services Hospital, Lahore, Pakistan; 3 Gul e Raana, FCPS. Department of Obstetrics and Gynaecology, Services Hospital, Lahore, Pakistan; 4 Anaab Wasim, MBBS Student. Lahore Medical & Dental College, Lahore, Pakistan

**Keywords:** Delays, Maternal mortality, Maternal mortality ratio (MMR), Postpartum hemorrhage

## Abstract

**Objective:**

To assess the maternal mortality ratio (MMR), causes, trends and delays in care over a period of twelve years at institutional level.

**Methods:**

This cross-sectional study was conducted over a 12 years period (2011-2022) at Services Hospital, Lahore, Pakistan. Maternal mortality ratio was calculated as number of maternal deaths per 100,000 live births. The study period was divided in two equal halves i.e. 2011-2016 and 2017-2022. Causes, trends in maternal mortality and delays were analyzed and compared between the two study periods. SPSS-23 was used for data analysis.

**Results:**

There were 222 maternal deaths and 47,853 live births during the study period. The cumulative MMR was 463.92/100,000 live births with a decreasing trend over 12 years. Postpartum hemorrhage and cardiac disease in pregnancy were leading cause of death in 44(31.65%) and 31(22.3%) of patients in 2011-2016, while hypertensive disorders in 18(21.69%) patients and sepsis in 15(18.0%) patients were significantly responsible for maternal mortality in 2017-2022 (p=0.00). Analysis of 12 years data showed first delay i.e. delay in seeking care was the commonest seen in 92(41.44%) patients, followed by second delay in 29(13.06%) and third delay in 27(12.16%) patients. Postpartum period and first delay were significantly associated with maternal mortality (p=0.05 and <0.001).

**Conclusion:**

Reduction in maternal mortality is a great challenge for developing countries like Pakistan. Although maternal mortality has declined over period of twelve years in our study, targeted interventions at community level are needed to address the delay in seeking care and improve maternal outcomes

## INTRODUCTION

Maternal mortality is not only a devastating event in the life of a family but is a strong predictor of growth, progress and development of a country. Globally MMR has declined by 34.3%(from 339 to 223 per 100,000 live births) between 2000 and 2020 with region of Southern Asia having the greatest percentage reduction in MMR of 67.5%(from 397 to 129).^1^ Analysis of trends in maternal mortality from 2000 to 2017 showed that Pakistan was amongst the ten countries which accounted for 60% of maternal deaths during this period.^2^ MMR survey conducted in 2019 by National institute of Population studies (NIPS) in Pakistan reported MMR of 186/100,000.^3^ Eliminating preventable maternal deaths remains important goal internationally. However, to fulfill the sustainable development goals, it needs to go further down to 70/100,000 live births by 2030.^4^

Most (80-85%) of maternal deaths in developing countries are due to direct causes (complications during pregnancy, childbirth and puerperium) including hemorrhage, sepsis, hypertensive disorders and complication of abortion while indirect causes ( due to pre existing maternal conditions which get aggravated by pregnancy) are responsible for 20-25% of maternal deaths 5,6 Most of these maternal deaths are preventable by improving the healthcare services, addressing the needs of patients across the continuum of care around the time of birth. High number of maternal deaths reflects the poor quality of healthcare system and timely unavailability of existing healthcare facilities to patients. Although coverage of health care has improved but social, economic and cultural barriers play a significant role in accessing the healthcare in low middle income countries (LMIC).

The ‘three delays’ model developed by Thaddeus and Maine is the most common framework used to evaluate circumstances surrounding maternal health.^7^ The first delay 1) is delay in decision to seek care, 2) is the delay in reaching a healthcare facility, and 3) is the delay in receiving timely and appropriate care at the health care facility. First delay is shaped by factors affecting decision-making, including sociocultural influences, financial constraints, and opportunity costs.

On the other hand, second delay arise from issues such as the distance to the nearest healthcare facility, travel time, transportation availability and cost, and road conditions. Reaching a health facility does not necessarily mean the end of the journey as the nearest facility may not be equipped to treat the condition or even administer essential first aid so patients are referred to another facility that is better equipped. The third delay relates to inadequacies within the health care system itself. This could be lack of properly trained personal, transfusion facility, equipment and other infrastructural inadequacies. Late or wrong diagnosis and incorrect action by the staff are other factors that contribute to delays in the timely provision of needed care. The three delays model helps to determine where improvements can be made to save the lives of women and babies.^8^

The studies in different parts of Pakistan have reported different MMR and the causative factors for maternal death.^9^,^10^ Reliable and timely data identifying the causes and factors responsible for the deaths is crucial for policy makers to plan effective interventions in right direction. Maternal mortality data is collected from various sources including household surveys, mortality studies, verbal autopsies, confidential enquiries and national surveys. Facility based data offers a contextualized resource for clinical and organizational quality improvement and inform local policy makers to target deficiencies in health systems.

Hence, a longer duration, maternal mortality review starting from 2011 to 2022 was planned. This study aimed to study factors associated with maternal deaths, causes of maternal death and evaluate site specific trends in the MMR over twelve-year time. To our knowledge, this study provides the latest data on MMR from a government tertiary care hospital that will aid in evaluating healthcare system efficiency and policy-making related to maternal mortality.

## METHODS

This study was conducted in Obstetrics department, Services Hospital, Lahore which is a public sector tertiary care hospital. This study had a review of all maternal deaths occurring in the from 1^st^ Jan 2011 to Dec 2022.

### Ethical Approval:

It was obtained from Institutional Review Board of Services Institute of Medical Sciences, IRB/2018/480/SIMS, dated November 24, 2018.

Data collection was done through a structured proforma and information was collected from labour room register, case notes of patient and maternal mortality registers. All maternal deaths in our department were included. A maternal death was defined as death of woman during pregnancy or within 42 days of termination of pregnancy from any cause related or aggravated by the pregnancy or its management.^1^ The number of live births occurring during this study period were calculated from yearly statistics. Maternal mortality ratio was calculated as number of maternal deaths per 100,000 live births. The study period was divided in two equal halves i.e. 2011-2016 and 2017-2022. Causes, trends in maternal mortality and three delays were analyzed and compared between the two study periods.

Descriptive statistics in the form of means, frequencies and percentages were calculated and statistical analysis in the form of paired t-test and regression analysis was conducted by using SPSS 23. P values were calculated to show the statistically significant difference. A p-value of less than 0.05 was considered as significant.

## RESULTS

The twelve years review of maternal mortality at a tertiary care hospital revealed distinct demographic trends. The mean age of women in this group was 28.87 ± 5.102 years, women aged 25 to 35 years constituted the highest proportion of cases 148 (66.7%), followed by 58(26.1%) under 25 years. Most of the women 150 (67.6%) were multigravida. The educational backgrounds varied, with a higher portion being either illiterate 74(33.3%) or having primary education 79 (35.6%). Alarmingly,135 (60.8%) of cases were un-booked for antenatal care in our hospital, and 87(39.2%) of women were not receiving any kind of antenatal care. A notable proportion 143 (64.4%) of cases came from the low socioeconomic class (Table-I).

**Table-I:** Characteristics of the Patients enrolled in the study.

Characteristics		N (%)
Age (years)	Mean age	28.87 ± 5.102
	<24	58(26.1%)
	25-35	148(66.7%)
	>35	16(7.2%)
Parity	Primigravida	72 (32.4%)
	Multigravida	150 (67.6%)
Education	Illiterate	74 (33.3%)
	Primary	79 (35.6%)
	Middle	35 (15.8%)
	Matric	34 (15.3%)
Booking	Un-booked	135 (60.8%)
	Booked	87 (39.2%)
Socioeconomic	Lower class	143 (64.4%)
	Middle class	71 (32%)
	High class	8 (3.6%)
Antenatal Care	Traditional birth attendants (TBAS)	86 (38.7%)
	Tertiary care hospital	47 (21.2%)
	No	89 (40.1%)

**Fig.1:**
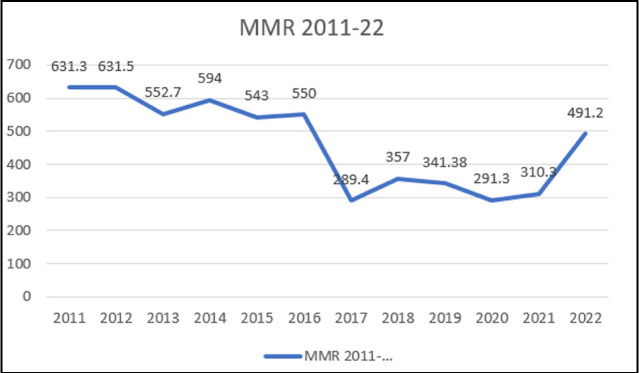
Trends in maternal mortality.

The results of our analysis show MMR was notably high in 2011 at 631.31, gradually declined over the years, reaching its lowest point in 2020 at 291.3 followed by a sharp rise in 2022 (Fig.1). Regarding causes of maternal mortality, direct causes accounted for 165(74.3%) deaths. Out of these direct causes the main causes were hemorrhage 65(29.2%) followed by hypertensive disorders 43(19.36%) and sepsis 36(16.21%). Indirect causes of mortality were 57((25.6%) out of which leading one was cardiac disease 40(18.0%) followed by infections (hepatitis and COVID)12 (5.4%).

For comparison of data the study period was divided in two groups i.e. 2011-2016 & 2017-2022. Postpartum haemorrhage and cardiac disease in pregnancy were leading cause of death in 44(31.65%) and 31(22.3%) of patients in 2011-2016, while hypertensive disorders in 18(21.69%) patients and sepsis in 15(18.0%) patients were significantly responsible for maternal mortality in 2017-2022 (p=0.00). No statistically significant difference was found between two groups in mode of delivery although caesarean section rose in subsequent years(p=0.380). However, causes of maternal mortality showed a significant decline (p<0.05) in last six years along with decrease in MMR. Maximum number of patients died in first 24 hours, 91(65.46%) in first period (2011-2016) vs 36(43.37%) in second one (2017-2022) p value <0.05(Table II)

**Table-II:** Comparison of maternal mortality trends between two periods, (2011-2016) & (2017-2022).

Variables		2011-2022 N=222	2011-2016 N=139	2017-2022 N=83	P-value
Time of death	Antepartum	34(15.31%)	21(15.11%)	13(15.66%)	0.462
	Intrapartum	13(58.55%)	8(5.76%)	5(6.02%)	
	Postpartum	152(68.46%)	99(71.22%)	53(63.86%)	
	Post abortion	23(10.36%)	11(7.91%)	12(14.46%)	
Cause of death	Postpartum hemorrhage	58(26.12%)	44(31.65%)	14(16.87%)	.000
	Placental abruption	7(3.1%)	2(1.44%)	5(6.02%)	
	Hypertensive disorders	43(19.36%)	25(17.99%)	18(21.69%)	
	Pulmonary embolism	21(9.45%)	12(8.63%)	9(10.84%)	
	Sepsis	36(16.21%)	21(15.1%)	15(18.0%)	
	Cardiac disease	40(18.0%)	31(22.3%)	9(10.8%)	
	Hepatitis A /E and COVID	12(5.4%)	4(2.87%)	8(9.6%)	
	Blood transfusion and Anaphylactic shock	5(2.25%)	2(1.43%)	3(3.6%)	
Mode of delivery	SVD	72(32.43%)	51(36.69%)	21(25.3%)	0.380
	LSCS	93(41.89%)	56(40.28%)	37(44.57%)	
	Abortion	23(10.36%)	11(7.91%)	12(8.6%)	
	Antepartum	34(15.31%)	21(15.10%)	13(15.66%)	
Three Delays Admission to death interval	1st delay	92(41.44%)	57(41.01%)	35(42.17%)	
	2nd delay	29(13.06%)	18(12.95%)	11(13.25%)	0.002
	3rd delay	27(12.16%)	8(5.76%)	19(22.89%)	
	Within 24 hours	91(65.46%)	36(43.37%)	127(57.20%)	0.001
	25 to 72 hours	57(25.67%)	26(18.70%)	31 (37.34%)	
	73 hours to 1 week	21(9.45%)	12(08.63%)	09 (10.84%)	
	>1week to 6 weeks	17(7.65%)	10(07.19%)	07 (08.43%)	

**Fig.2:**
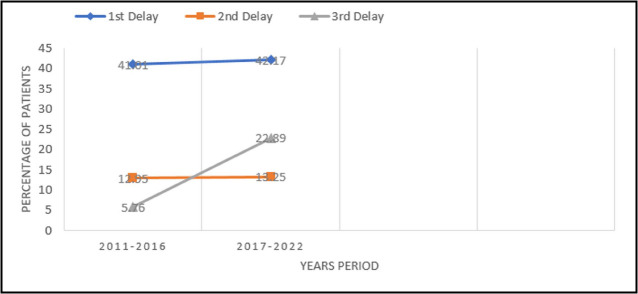
Trends of three delays of care over two periods of years.

Using three delays of seeking care as a framework for assessing maternal mortality, Analysis of 12 years data showed that first delay was the commonest 92(41.44%) followed by second 29(13.06%) and third delay 27(12.16%). Comparison of two halves of the study period showed no significant change in 1^st^ & 2^nd^ delay however third delay substantially increased from 8 (5.76%) to 19 (22.89%) (p=0.002) (Fig 2). After application of regression analysis, the present study highlighted that time of death (postpartum) was significantly associated with maternal mortality. The women experiencing the delays were nine times more likely to have mortality compared to the women who were not facing the delays, (Table-III).

**Table III:** Regression analysis of factors affecting maternal mortality

Predictors	Odds ratio	CI 95%	p-value
Time of death(postpartum)	0.625	0.384 - 1.018	0.059
Mode of delivery	1.141	0.856 - 1.522	0.367
First Delay	9.777	2.66 - 35.87	< 0.001

OR: Odds ratio; CI: Confidence Interval; p≤0.05 considered significant.

## DISCUSSION

Our study reports cumulative MMR of 463.92/100,000 live births. Mean age of the cases of maternal deaths was 28.87 ± 5.102 years and majority had two to three children (multigravida). Review of maternal mortalities from other tertiary care hospitals in Pakistan shows similar data.^9^,^10^ Most of these women were uneducated, belonged to lower income group and they did not have access to basic health needs. These characteristics are similar in maternal deaths reported from other LMIC.^5^-^8^ Girl education is powerful tool that can empower them to make informed decision about their sexual and reproductive health.

There is a decreasing trend seen in MMR till 2021 followed by rise in 2022. Declining trends in MMR in our study till 2021 is consistent with a retrospective study from Hyderabad, Sindh reported comparatively higher cumulative MMR of 1521.5/100,000 live births with a downward trend over 20 years.^10^ Another study from Peshawar reported MMR of 431/100,000 live births with decreasing trend from 2013-2017.^11^ There is an overall decline in maternal mortality reflecting impact of various attempts of the Punjab government to improve the healthcare facilities regarding antenatal care and delivery by skilled birth attendants. The number of skilled birth attendants has increased from 26% in 1991 to 91% in 2019 and the number of deliveries at these facilities has also increased from 14 to 71% during this period.^12^ However, MMR estimation done from tertiary care hospital show high number as majority patients with complications are referred there from lower levels of care emphasizing the need for continuous monitoring and targeted interventions to sustain and improve maternal health outcomes at tertiary care hospitals.^13^

There is a rise in MMR in the year 2022. This may be due to combination of different factors: Rising rate of C-section leading to increase in number of cases of morbidly adherent placenta leading to antenatal, intrapartum and post-partum complications^11^ and implementation of MPDSR system (Maternal and perinatal death surveillance response system) in the department. MPDSR is a dependable tool introduced by WHO to identify, quantify and prevent maternal and perinatal death in countries with no uniform policy to report and analyze maternal deaths. The primary target of MPDSR is reducing future avoidable maternal mortality through a continuous action and surveillance cycle followed by the interpretation of the collected information to recommend actions that will prevent future deaths.^14^

Postpartum hemorrhage, hypertensive disorders, cardiac diseases and sepsis were the most common causes of maternal mortality in our study. Similar results were shown in previous studies as well.^4^-^12^,^15^ Our study showed a significant decline in incidence of deaths due to post-partum hemorrhage from 2012 to 2022. Although hemorrhage remains leading cause of deaths worldwide, incidence of uterine atony has reduced globally because of trained healthcare professionals’ availability during childbirth leading to early recognition and management of uterine atony.^16^ Increased use of uterotonics like misoprostol along with standardized guidelines and protocols for managing postpartum hemorrhage (PPH) also has a role to play.

The majority of maternal deaths occurred in postpartum period in our study which is actual crucial period of surveillance, similar to other studies.^5^,^6^,^8^-^10^ Proper training of HCPs and awareness of families should be done to address and prevent avoidable factors.

The three delays model takes a holistic approach to understanding the various responsibilities at the household, community, and health system levels to prevent maternal deaths. The majority of our patients had first delay (41.44%) as more than half deaths occurred within 24 hours of admission, which is failure to recognize the early signs of complications and decision to seek help. Similar data is reported in other studies from Pakistan.^10^-^12^ The pregnant ladies often delay in seeking necessary health care because of lack of education, autonomy, empowerment and poor socioeconomic condition. Women are usually dependent on their husband to decide about their medical care, in addition to that these ladies feel that care of others at home is more important for them.^17^ Illiteracy, lack of antenatal care and poor socioeconomic class were important factors associated with maternal mortality in our study as is reported from other studies of LMIC.^7^,^8^,^18^ Almost 60% of the women who died, did not have any antenatal booking in our hospital and 39% of them did not had any antenatal booking in any healthcare setup. Every woman requires access to superior healthcare throughout pregnancy, childbirth, and the postpartum period. Maternal well-being is intricately connected to newborn health, underscoring the critical necessity of skilled healthcare professionals attending all births. Timely intervention and care from trained professionals can significantly impact the survival outcomes for both mothers and newborns.^19^

Once women decide to seek care, there is usually much delay in reaching healthcare facility which can provide appropriate care representing second delay. Lack of funds, non-availability of transport and infrastructure are prime reasons.^20^,^21^ Third delay in our study has been reported to be around 12% however it is much higher (96.8%) in studies from Malawi and Egypt.^8^,^22^ Lack of proper care at healthcare facility due to non-availability of required resources, inadequate blood transfusion services, dearth of qualified personals and equipment are most important reasons for deaths despite reaching a facility.

Maternal mortality reduction is a great challenge for government to meet SGD target. There is a great disparity in access between rural and urban populations, with the majority of Pakistan’s citizens residing in rural regions where healthcare infrastructure is sparse.^23^ To address these challenges, it is imperative for governments and organizations to prioritize funding allocations, particularly in rural areas, to train more qualified healthcare professionals, establish additional healthcare facilities, and raise awareness about the importance of antenatal, delivery, and postpartum care.

### Limitations:

Since we collected data from a tertiary care hospital in Punjab, this may limit its generalizability to other provinces of Pakistan due to different healthcare systems. Our study highlighted trends in maternal mortality but being a cross-sectional study, it may not provide direct insights into the effectiveness of specific interventions aimed at reducing maternal mortality rates. Future research should explore this area.

## CONCLUSION

Reduction in maternal mortality is a great challenge for developing countries like Pakistan. Changing maternal health dynamics over the twelve-year period emphasizes the need for continuous monitoring and targeted interventions to sustain and improve maternal health outcomes at the hospitals.

## References

[1] Trends in maternal mortality 2000 to 2020: Estimates by WHO, UNICEF, UNFPA, World Bank Group and UNDESA/Population Division [Internet] World Health Organization.

[2] Trends in maternal mortality: 2000 to 2017 [Internet] https://www.unfpa.org/featured-publication/trendsmaternal-mortality-2000-2017.

[3] NIPS P (2019). Pakistan Maternal Mortality Survey 2019- Key Indicators Report.

[4] World Health Organization (2016). World health statistics 2016:monitoring health for the SDGs sustainable development goals.

[5] Lawrence ER, Klein TJ, Beyuo TK (2022). Maternal Mortality in Low and Middle-Income Countries. Obstet Gynecol Clin North Am.

[6] Kassebaum NJ, Barber RM, Bhutta ZA, Dandona L, Gething PW, Hay SI (2016). Global, regional, and national levels of Maternal Mortality, 1990–2015:A systematic analysis for the global burden of disease study 2015. Lancet.

[7] Thaddeus S, Maine D (1994). Too far to walk:maternal mortality in context. Soc Sci Med.

[8] Mgawadere F, Unkels R, Kazembe A, Van den Broek N (2017). Factors associated with maternal mortality in Malawi:Application of the three delays model. BMC Pregnancy and Childbirth.

[9] Bano N, Chaudhri R, Yasmeen L, Shafi F, Ejaz L (2011). A study of maternal mortality in eight principal hospitals in Pakistan in 2009. Int J Gynecol Obstet.

[10] Nisar N, Abbasi RM, Chana SR, Rizwan N, Badar R (2017). Maternal Mortality In Pakistan:Is There Any Metamorphosis Towards Betterment?. J Ayub Med Coll Abbottabad.

[11] Rafiq S, Syed W, Ghaffar SF (2019). Trends and causes of maternal mortality in a tertiary care hospital over five years: 2013-2017. Pak J Med Sci.

[12] Zafar AF, Javed N, Niaz A, Noor S, Tahira T (2019). Maternal Mortality:Interesting Comparison between Tertiary, Secondary and Primary Centers in Pakistan. Ann King Edward Med Univ.

[13] Majeed N, Akhtar R (2019). Provision of Round the Clock Basic Obstetric and Neonatal Care Services in Rural Settings:A low cost, high impact intervention in Punjab. Ann King Edward Med Coll.

[14] Pegu B, Thiagaraju C, Nayak D, Subbaiah M (2021). Placenta accreta spectrum-a catastrophic situation in obstetrics. Obstet Gynecol Sci.

[15] Arif A, Alam MB (2023). Maternal and perinatal death surveillance and response in Baluchistan, Pakistan-causes &contributory factors of maternal deaths. Population Med.

[16] Jena BH, Biks GA, Gete YK, Gelaye KA (2023). Determinants of postpartum uterine atony in urban South Ethiopia a community-based unmatched nested case–control study. BMC Pregnancy Childbirth.

[17] Health Services Academy and the National Ministry for Health Services Coordination and Regulation Improving Maternal, Newborn and Child Health Outcomes in a Decade [Internet 2020].

[18] Bailey PE, Andualem W, Brun M, Freedman L, Gbangbade S, Kante M (2017). Institutional maternal and perinatal deaths:A review of 40 low and middle income countries. BMC Pregnancy Childbirth.

[19] Kebede TT, Godana W, Utaile MM, Sebsibe YB (2021). Effects of antenatal care service utilization on maternal near miss in Gamo Gofa zone, southern Ethiopia:retrospective cohort study. BMC Pregnancy Childbirth.

[20] Shaeen SK, Tharwani ZH, Bilal W, Islam Z, Essar MY (2022). Maternal mortality in Pakistan:Challenges, efforts, and recommendations. Ann Med Surg.

[21] Wasim T, Wasim M, Mushtaq J, Amin Z, Asghar S (2021). Maternal near-miss, mortality and their correlates at a tertiary care hospital. J Pak Med Assoc.

[22] Mohammed MM, El Gelany S, Eladwy AR, Ali EI, Gadelrab MT, Ibrahim EM (2020). A ten year analysis of maternal deaths in a tertiary hospital using the three delays model. BMC Pregnancy Childbirth.

[23] Aziz A, Saleem S, Nolen TL, Pradhan NA, McClure EM, Jessani S (2020). Why are the Pakistani maternal, fetal and newborn outcomes so poor compared to other low and middle-income countries?. Reprod Health.

